# miRNA expression and interaction with the 3′UTR of FMR1 in FRAXopathy pathogenesis

**DOI:** 10.1016/j.ncrna.2020.11.006

**Published:** 2020-12-03

**Authors:** Alexander A. Dolskiy, Andrey A. Yarushkin, Irina V. Grishchenko, Natalya A. Lemskaya, Alexey V. Pindyurin, Lidiya V. Boldyreva, Vladimir O. Pustylnyak, Dmitry V. Yudkin

**Affiliations:** a State Research Center of Virology and Biotechnology “Vector”, Federal Service for Surveillance on Consumer Rights Protection and Human Well-being (FBRI SRC VB “Vector”, Rospotrebnadzor), Koltsovo, Novosibirsk Region, Russia; b Federal Research Center of Fundamental and Translational Medicine, Novosibirsk, Novosibirsk Region, Russia; c Novosibirsk State University, Novosibirsk, Novosibirsk Region, Russia; d Institute of Molecular and Cellular Biology, Siberian Branch of the Russian Academy of Sciences, Novosibirsk, Novosibirsk Region, Russia

**Keywords:** miRNAs, 3′UTR, FMR1, Fragile X syndrome, Fragile X associated tremor/ataxia syndrome, Fragile X-associated primary ovarian insufficiency

## Abstract

FRAXopathies are caused by the expansion of the CGG repeat in the 5′UTR of the *FMR1* gene, which encodes the protein responsible for the synthesis of FMRP. This mutation leads to dramatic changes in FMRP expression at both the mRNA and protein levels. Evidence is emerging that changes in *FMR1* mRNA expression can lead to the dysregulation of the miRNAs that target its 3′UTR. In the present work, B-lymphocyte cell lines obtained from patients with FRAXopathies were used, and a wide variety of *FMR1* gene activities were observed, allowing the identification of the relationships between *FMR1* dysregulation and miRNA activity. We studied the expression levels of eight miRNAs that target the *FMR1* gene. To prove the interaction of the studied miRNAs with *FMR1*, a plasmid was constructed that possesses three primary structures: the miRNA gene, with expression driven by an inducible promoter; a constitutively expressed FusionRed reporter; and an eGFP reporter followed by the 3′UTR of the *FMR1* gene. We evaluated changes in miRNA expression in response to alterations in *FMR1* gene activity in a model cell line as well as interactions with some miRNAs with the *FMR1* 3′UTR.

## 1 Introduction

FRAXopathies include three syndromes that develop due to the expansion of the CGG repeat in the 5′UTR of the *FMR1* gene, which encodes FMRP [1]. The first disorder, fragile X syndrome (FXS; OMIM # 300624), is the most common form of inherited intellectual disability, with population frequencies of approximately 1/4000 and 1/6000 in men and women, respectively, values that vary in different countries [2]. The second disorder, fragile X-associated tremor/ataxia syndrome (FXTAS; OMIM # 300623), is a progressive neurodegenerative disturbance characterized by kinetic tremors, cerebellar ataxia, parkinsonism, neuropathy, and executive dysfunction [3,4]. The third disorder, fragile X-associated primary ovarian insufficiency (FXPOI; OMIM # 311360), is a syndrome characterized by a decrease in the frequency or amplitude of gonadotropin secretion and early menopause before the age of 40 [5]. The normal *FMR1* allele is characterized by fewer than 55 CGG repeats, resulting in optimal amounts of mRNA and protein being synthesized. With the expansion of the number of CGG triplets from 55 to 200, the *FMR1* allele is considered to be in a state of premutation [6]. The frequencies of occurrence of this gene variant are 1/200 to 1/300 in women and 1/250 to 1/450 in men [7]. Premutation is associated with FXTAS and FXPOI and is accompanied by changes in *FMR1* gene expression at the mRNA level, resulting in a concurrent decrease in protein levels [8]. Further expansion of the CGG repeat to more than 200 copies leads to the methylation of the *FMR1* gene promoter region and the absence of FMR1 mRNA and FMRP, resulting in the manifestation of FXS [6].

Notably, the manifested symptoms between full mutation and premutation carriers are different, indicating different mechanisms of pathogenesis. In the case of FXS, this mechanism involves the absence of FMRP [9], while for premutation syndromes, the development of pathologies is associated with changes in *FMR1* mRNA and protein levels [10]. One of the reasons for the development of premutation syndromes involves a gain-of-function mechanism [11]. In this case, *FMR1* mRNA levels significantly exceed normal levels, and the excess mRNA binds to proteins in the nucleus. A region of mRNA with CGG repeats binds to one or more CGG-binding proteins and forms inclusions, thereby isolating these proteins and blocking their cellular functions [12]. These inclusions contain proteins such as Sam68 and DGCR8, which are involved in mRNA splicing and transport and are also involved in the maturation of a number of miRNAs [13]. Another reason for the development of premutation syndromes involves disrupted miRNA pathways. At present, increasing evidence indicates that miRNAs can be involved in the manifestation of FRAXopathies [14]. These miRNAs can be divided into two groups, the first of which includes those that interact directly with FMRP. It was previously shown that FMRP, interacting with miR-125b, suppresses the expression of the NR2A protein, a subunit of the NMDA receptor that affects synaptic plasticity [15]. FMRP and miR-181d have also been shown to coregulate axon elongation by suppressing the translation of Map1, a protein associated with microtubules, and Calm1, a regulator of calcium signaling. After stimulation with a growth factor, Map1 and Calm1 are released from granules suppressed by FMRP and miR-181d and participate in translation during axon lengthening [16]. The second group of miRNAs includes those that directly interact with *FMR1* mRNA and can be dysregulated in response to gene activity. The primary target for miRNAs is the 3′UTR of genes [17]. Because miRNAs have many target genes, changes in their activity in response to changes in gene expression can lead to the dysregulation of other genes, resulting in the development of diseases and a multiplicity of symptoms [18]. In a mouse model of FXTAS, complementarity was demonstrated between the 3′-untranslated region of *Fmr1* mRNA and miR-101, miR-129-5p, and miR-221, which are expressed in nervous tissue. The results of this study revealed that miR-221 expression was reduced by 40% in the brain tissues of model mice compared to that observed in healthy controls. In addition, the overexpression of this miRNA in fibroblasts obtained from a patient with FXTAS resulted in a significant decrease in *FMR1* mRNA compared to that observed in the control cell line [14]. It is also assumed that miR-130b is a regulator of *Fmr1* expression and is responsible for neural progenitor cell determination [19]. Thus, the degree of symptom manifestation may be associated with the influence of miRNAs, the expression levels of which are altered in response to changes in *FMR1* gene expression. Alterations in miRNA levels lead to changes in the expression of other target genes that are associated with many FRAXopathy symptoms.

The goal of the present study was to investigate the dysregulation of miRNA expression in response to changes in *FMR1* gene expression. The eight miRNAs that target the *FMR1* gene were investigated. We used B-lymphocyte cultures obtained from patients with FRAXopathies that were grouped by *FMR1* gene activity and determined the correlations between miRNA and *FMR1* expression in these groups. The miRNAs presumably interacting with the 3′UTR of *FMR1* were identified in open databases based on the target score value. Their interaction with that region of mRNA was experimentally confirmed. To this end, a new model plasmid was constructed consisting of an miRNA gene driven by an inducible promoter, a constitutively expressed FusionRed reporter and an eGFP reporter gene linked to the 3′UTR of *FMR1*. In this system, when miRNA interacts with the target sequence, the eGFP level decreases. Using this approach, we investigated the interaction of the selected miRNAs with the 3′UTR of *FMR1* to evaluate their involvement in the development of FRAXopathies.

## 2 Results

### 2.1 Cell line classification

It is generally believed that in FMR1 premutation carriers, mRNA expression increases, but FMRP levels decrease [10]. However, studies on cell cultures from patients have shown that this is not always the case. Because miRNAs act at the posttranscriptional level to regulate protein expression, to analyze their expression, we decided to classify cell lines by the ratio of *FMR1* mRNA expression and the level of FMRP. The levels of FMR1 mRNA and FMRP were measured in every cell line relative to those in the GM06895 normal cell line.

In this study, immortalized B lymphocytes from patients with FRAXopathies were used as model cell lines. To analyze *FMR1* mRNA expression, total RNA was isolated from unsynchronized cell cultures, and the expression level was analyzed by real-time PCR (Table 1). The expression level was measured in three biological and three technical repetitions for each sample. The results showed that in the GM06865 cell line carrying the normal *FMR1* gene allele, the expression of this gene was 1.17-fold higher than that observed in the GM06895 cell line. *FMR1* gene expression in the GM06891 cell line carrying the premutant allele was 2.07-fold higher than that observed in the GM06895 cell line. In the GM06891E cell line with a large premutation, *FMR1* expression was 0.89-fold that observed in the normal GM06895 cell line and was not significantly different. *FMR1* mRNA expression levels in the CPG8, CPG166 and GM06897 cell lines were 0.64-, 0.85- and 0.7-fold lower than that observed in the GM06895 cell line, respectively, all of which represented significant differences (P < 0.05). The analysis of the *FMR1* promoter region using a methylation-sensitive restriction endonuclease did not reveal complete methylation in these cell lines, although this approach cannot detect the partial methylation of this region, which is probably a factor in reducing transcription. *FMR1* expression in a CPG10 cell line with a premutation allele was 1.66-fold higher than that observed in the GM06895 cell line. In three cell lines obtained from patients with FXS (GM04025, CPG18 and CPG7), *FMR1* mRNA was not detected.

**Table 1:** Cell line groups.

Group	Cell lines	S.	Meth.	Allele	Relative signal level of FMRP protein	Relative level of *FMR1* mRNA	Separation criterion FMRP/*FMR1* mRNA	Characteristics of cell groups
1	GM06865	M	absent	N^a^	0.3	1.17	0.26	Cell lines carrying normal and premutation alleles, with ratio values less than one
	GM06891	M	absent	PM^b^	0.71	2.07	0.34	
2	GM06891E	M	absent	PM (with somatic instability)	0.95	0.89	1.07	Cell lines carrying normal and premutation alleles, with ratio values greater than one
	CPG8	F	no data	N	0.87	0.64	1.36	
	CPG166	M	absent	N	1.27	0.85	1.49	
3	GM06897	M	absent	UFM^c^	4.56	0.7	6.5	Cell lines carrying unmethylated full mutation allele, with ratio values greater than one
4	CPG10	F	no data	N, PM	–	1.66	–	A cell line with a normal genotype without FMRP synthesis
5	GM04025	M	present	FM^d^	–	–	–	Cell lines with full mutation alleles without *FMR1* gene activity
	CPG18	M	present	FM	–	–	–	
	CPG7	M	present	FM	–	–	–	
	GM06895	M	absent	N	1	1	1	A control cell line

a N – Normal allele.

b PM – Premutation allele.

c UFM – Unmethylated full mutation allele.

d FM – Full mutation allele.

An analysis of the FMRP levels (Table 1) in the assayed cell lines showed that in the GM06891 line with a premutation allele, the protein level was 0.71-fold that observed in the control. In the GM06891E and CPG8 cell lines, FMRP expression at the protein and mRNA levels was lower than that detected in the GM06895 cell line. FMRP levels in the CPG166 and GM06897 cell lines were 1.27- and 4.56-fold higher than those observed in the GM06895 line, respectively, whereas *FMR1* mRNA expression in these cultures was reduced. In the CPG10 cell line with a premutation allele, which exhibits increased *FMR1* mRNA expression, FMRP protein was absent. In the GM04025, CPG7 and CPG18 lines, FMRP protein was also absent, which is typical for a full FMRP mutation.

Based on the ratio of the FMRP protein level and *FMR1* gene expression, which characterizes the activity of this gene, cell lines were divided into 5 groups (Table 1). Group 1 included two cell lines, GM06865 and GM06891, with normal and premutation alleles, respectively. Both cell lines showed FMRP/*FMR1* mRNA levels less than 1. Group 2 included three cell lines (GM06891E, CPG8, and CPG166) with normal and premutation alleles and FMRP/*FMR1* mRNA levels higher than 1. Group 3 contained only one cell line, GM06897, which harbors an unmethylated full mutation allele and is characterized by an FMRP/*FMR1* mRNA level that is much higher than 1. Group 4 included the CPG10 cell line, which has increased *FMR1* mRNA expression but an absence of FMRP protein expression. Group 5 includes the GM04025, CPG18 and CPG7 cell lines, which have full mutation alleles in which neither *FMR1* mRNA nor FMRP protein were detected.

### 2.2 Analysis of miRNA expression in the cell line groups

The analyzed miRNAs were selected using the mirBase.org, mirdb.org, and targetscan.org databases. The criteria for selection were known brain expression and complementarity to the 3′UTR of the *FMR1* gene with a target score of 79 or higher (Table S1). The participation of these miRNAs has been demonstrated in the development of both neurons and brain tumors [[20], [21], [22], [23], [24], [25]]. Based on this analysis, the following miRNAs were selected: hsa-miR-182-5p, hsa-miR-23a-3p, hsa-miR-25-3p, hsa-miR-148a-3p, hsa-miR-410-3p, hsa-miR-139-5p, hsa-miR-221-3p, and hsa-miR-302a-3p. The role of hsa-miR-23a-3p in the function of neurons is not known, which does not demonstrate its absence, only the lack of sufficient information.

**Fig. 1:**
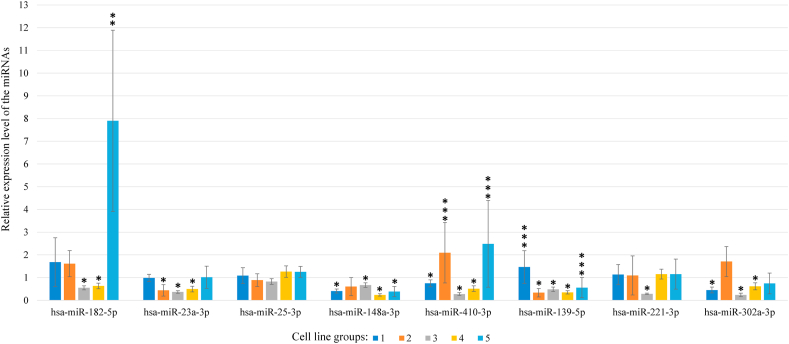
**Relative expression levels of the studied miRNAs.** (*) Significant decrease in miRNA level relative to that in the GM06895 control cell line, P < 0.05; (**) Significant increase in miRNA level relative to that in the GM06895 control cell line, P < 0.05; (***) Tendency of change in miRNA level relative to that in the GM06895 control cell line, P > 0.05.

After the cell lines were divided into groups, miRNA expression was analyzed and is reported as the average value of all biological repeats (Fig. 1).

*hsa-miR-182-5p*: In groups 1 and 2, hsa-miR-182-5p expression was unchanged compared to that observed in the normal GM06895 cell line, whereas the expression levels observed in groups 3 and 4 were 0.54- and 0.63-fold lower (P < 0.05), while expression was increased by 7.9-fold in group 5 (P < 0.05).

*hsa-miR-23a-3p*: In groups 1 and 5, hsa-miR-23a-3p expression was unchanged compared to that observed in the GM06895 cell line, while the expression levels observed in groups 2, 3, and 4 were 0.43-, 0.36- and 0.49-fold lower, respectively.

*hsa-miR-25-3p*: The levels of hsa-miR-25-3p expression were unchanged in all cell groups.

*hsa-miR-148a-3p*: In groups 1, 3, 4, and 5, hsa-miR-148a-3p mRNA levels were significantly decreased by 0.4-, 0.67-, 0.24- and 0.39-fold compared to those observed in the GM06895 cell line, respectively, while no significant change was observed for group 2.

*hsa-miR-410-3p:* In groups 1, 3 and 4, hsa-miR-410-3p was decreased by 0.75-, 0.27- and 0.51-fold compared to that observed in the GM06895 cell line, whereas the levels observed in groups 2 and 5 were increased by 2.09- and 2.49-fold, although these latter differences were not significant (P > 0.05).

*hsa-miR-139-5p*: In group 1, a tendency toward increased hsa-miR-139-5p expression was observed compared to that detected in the GM06895 cell line, although this difference was not significant (P > 0.05). In groups 2, 3 and 4, hsa-miR-139-5p expression was decreased by 0.33, 0.48 and 0.35 compared to that observed in the GM06895 cell line, respectively (P < 0.05). A decreasing trend for hsa-miR-139-5p expression was also observed for group 5, although the difference was not significant (P > 0.05).

*hsa-miR-221-3p*: No difference in hsa-miR-221-3p was observed between groups 1, 2, 4, and 5 and the control cell line, while a 0.28-fold decrease in expression was observed for group 3 (P < 0.05).

*hsa-miR-302a-3p*: In groups 1, 3 and 4, hsa-miR-302a-3p expression was decreased by 0.45-, 0.24- and 0.61-fold compared to that observed in the GM06895 cell line, respectively (P < 0.05), while no significant differences in expression were observed for groups 2 and 5.

### 2.3 miRNA interaction analysis

**Fig. 2:**
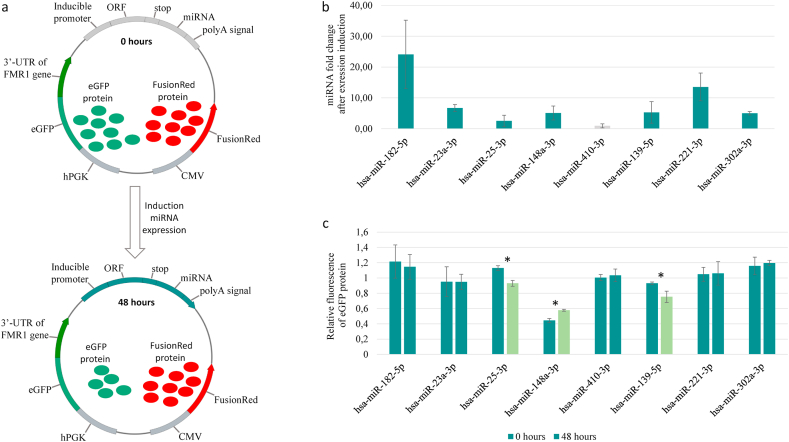
(a) **Scheme for the generation of the constructed plasmid.** At the initial timepoint (0 h), mRNAs encoding eGFP and FusionRed proteins are transcribed from two constitutive promoters (hPGK and CMV). When doxycycline is added, the inducible promoter is activated, and the microRNA is synthesized and processed into a mature form, leading to its interaction with the 3′UTR of the *FMR1* gene present in the eGFP mRNA, which ultimately reduces the amount of eGFP protein generated. (b) **Analysis of the induction of mature forms of miRNAs in genetic constructs.** The results are presented as fold changes in miRNA expression after 48 h of induction relative to the values observed at the initial time point. (c) **Analysis of the interactions of miRNAs and the 3′UTR of *FMR1***. Relative expression level of the reporter protein before (0 h of doxycycline treatment) and after (48 h of doxycycline treatment) microRNA expression induction. (*) Significant differences, where P < 0.05.

The studied miRNAs were identified in open databases based on target score value. Their interactions with the 3′UTR of the *FMR1* gene were predicted by bioinformatic analysis in these resources [[26], [27], [28], [29]]. Thus, experimental evidence is required to confirm these interactions. To this end, a model plasmid was constructed (Fig. 2a) encoding two reporter proteins (eGFP and FusionRed) expressed from constitutive promoters (hPGK and CMV, respectively). In this plasmid, the 3′UTR of the *FMR1* gene is located downstream of the eGFP ORF, and the miRNA gene is driven by a tetracycline-inducible promoter. Between the puromycin resistance ORF and the SV40 transcriptional terminator, there is a Bpu14I restriction site at which different pre-miRNA sequences were cloned. This arrangement of the miRNA gene has been previously described [14] and is advantageous because translation does not occur during the synthesis of mRNA for the puromycin resistance gene from the region of the miRNA gene, which could interfere with the creation of secondary structures that are necessary for its proper maturation. If an miRNA interacts with the 3′UTR of *FMR1* mRNA, eGFP protein levels should change, whereas the level of FusionRed protein should remain constant. If there is no interaction between an miRNA and the 3′UTR, the expression levels of both reporter proteins should not change.

To test the efficiency of the created genetic constructs, the expression levels of the mature forms of the studied miRNAs were analyzed at the initial time point and at 48 h after the induction of expression with doxycycline (Fig. 2b). Because transient transfection with the created constructs was performed in the present study, constitutive reporter expression at the FusionRed mRNA level was used for normalization for accurate analysis of miRNA expression. The results showed that hsa-miR-182-5p, hsa-miR-23a-3p, hsa-miR-148a-3p, hsa-miR-139-5p, hsa-miR-221-3p and hsa-miR-302a-3p expression levels were increased by 24.11-, 6.71-, 5.11-, 5.32-, 13.52-, and 5.02-fold, respectively, after induction. An insignificant increase in miRNA expression (2.53-fold) was observed for the plasmid harboring the hsa-miR-25-3p gene, while no significant increase in hsa-miR-410-3p expression was detected after activation from an inducible promoter.

The fluorescence of the reporter proteins was measured on the first day after transfection before induction and after 48 h of incubation with doxycycline (Fig. 2c). No significant changes in reporter protein fluorescence were observed after the expression of hsa-miR-182-5p, hsa-miR-23a-3p, hsa-miR-410-3p, hsa-miR-221-3p, and hsa-miR-302a-3p. For hsa-miR-148a-3p, the reporter protein fluorescence was significantly increased from 0.44- to 0.58-fold after 48 h of induction. In cell lines transfected with plasmids harboring the hsa-miR-25-3p and hsa-miR-139-5p genes, the green protein signal significantly decreased (P < 0.05) after doxycycline induction from 1.13- to 0.93-fold and from 0.93- to 0.75-fold, respectively.

## 3 Discussion

In the present study, 8 miRNAs complementary to the 3′UTR of the *FMR1* gene were selected using mirBase.org, microRNA.org, and targetscan.org, with the majority of the selected miRNAs being involved in the development of neurons and brain tumors.

To study the differences in miRNA expression, we grouped cell lines by *FMR1* gene expression. miRNAs primarily regulate gene expression at the posttranscriptional level, and the ratio of *FMR1* mRNA to FMRP is more crucial for understanding this process than the CGG repeat size. Our results demonstrated that *FMR1* gene expression showed variability for each allele in cell cultures. We hypothesized that increasing miRNA expression may be associated with decreased FMRP levels and vice versa. To assess this possibility, cells were divided into 5 groups by *FMR1* activity relative to the control GM06895 cell line. Groups 1 and 2 included normal and premutant cell lines exhibiting altered *FMR1* mRNA and FMRP ratios. Group 5 harbors typical full mutations without *FMR1* gene expression. However, for groups 3 and 4, the allele type was considered for a separate cell line with an unusual genotype, such as GM06897, with an unmethylated full mutation, and CPG10, which has a heterozygous normal/premutation but does not express FMRP. All described changes in gene expression are not typical for each allele, but such unusual mRNA and FMRP expression profiles can promote a better understanding of miRNA dysregulation.

The analysis of miRNA expression in the described groups of cells revealed that the major tendency was decreased expression relative to that observed in GM06895 cells. Only one miRNA, hsa-miR-182-5p, showed a dramatic increase in group 5, the members of which have a typical full mutation and do not express FMRP. Most changes were observed for groups 3 and 4, which included cell lines with unusual genotypes (GM06897 and CPG10), and almost all studied miRNAs exhibited decreased expression in these cells. Minor changes were detected in group 2, the members of which have normal and premutant *FMR1* alleles.

Interestingly, the expression of hsa-miR-25-3p, with a target score of 94, did not change in any of the studied cells. The greatest changes in expression were observed for hsa-miR-23a-3p, hsa-miR-148a-3p, hsa-miR-410-3p, hsa-miR-139-5p and hsa-miR-302a-3p. Only one cell line, GM06897, exhibited greatly increased FMRP levels, and this was a cell line in which we showed changes in hsa-miR-221-3p expression. All of these changes may be due to the interaction of miRNAs with the 3′UTR of the *FMR1* gene, resulting in changes in *FMR1* activity or their own activity by other factors.

To investigate the potential interaction between the selected miRNAs and the 3′UTR of *FMR1* mRNA, we assembled genetically engineered constructs encoding two fluorescent reporter proteins (eGFP and FusionRed) under the control of constitutive promoters. The 3′UTR of the *FMR1* gene was cloned downstream of the eGFP ORF. The constructs also harbored a studied miRNA gene under the control of an inducible promoter. These plasmids were used to transiently transfect HEK293A cells, induce the expression of the encoded miRNA, and study the changes in the fluorescence of reporter proteins. In this model system, to determine the interaction of the investigated miRNAs with the 3′UTR of the *FMR1* gene, it is necessary to induce the overexpression of miRNA encoded into the construction. A decrease in the level of eGFP reporter protein coupled to the 3′UTR of the *FMR1* gene indicates their targeted interaction. Thus, this study sheds light on the direct interaction of the investigated miRNAs with the 3′UTR of the *FMR1* gene, while the study of their expression in cell cultures shows dysregulation in response to changes in the level of *FMR1* expression. Conclusions can be drawn only about the direct involvement of certain miRNAs based on the results of both experiments.

We analyzed the effectiveness of miRNA gene activation for all the generated plasmid constructs and observed miRNA expression for all plasmids except the plasmid containing the hsa-miR-410-3p gene. In addition, an insignificant increase in miRNA expression was observed for the plasmid bearing the hsa-miR-25-3p gene.

We observed a significant decrease in the fluorescence of the eGFP reporter protein coupled to the 3′UTR of the *FMR1* gene upon induction of miR-139-5p and miR-25-3p expression. As described above, the level of hsa-miR-25-3p expression did not change in the cell lines regardless of the genotype for the *FMR1* gene. Thus, this microRNA does not change its expression with different gene activities, although it has the ability to bind to the 3′UTR sequence. It is possible that this microRNA is involved in the regulation of this gene only at certain stages of ontogenesis, and its expression does not depend on *FMR1* gene CGG repeat length. Moreover, for miR-139-5p, a decrease in expression was observed in all cell lines except the normal one. For miR-302a-3p, miR-410-3p, miR-23a-3p, miR-221-3p, and miR-182-5p, changes in expression were demonstrated for different genotypes, but their interaction was not observed with the 3′UTR of the *FMR1* mRNA gene in the model plasmid. Interestingly, a significant increase in reporter protein was observed for the plasmid containing hsa-miR-148a-3p, indicating that it can serve as a translational activator, although this miRNA was previously shown only to suppress gene expression [30].

In summary, we have shown changes in expression for a number of miRNAs, but an interaction with the 3′UTR of the *FMR1* gene was observed for miR-139-5p only. The level of expression of this miRNA was decreased in all cell lines except the normal one, indicating its dysregulation during *FMR1* gene expression changes and, possibly, into pathogenesis in FRAXopathies. Two ways of recruiting this microRNA are suggested. First, microRNAs can change expression, which leads to a change in the level of the FMR1 protein. Second, a change in gene activity leads to a change in the expression of microRNAs that interact with it. Such an event could lead to a change in its other target genes. However, the search for causal relationships between these two events will require further research. Furthermore, other miRNAs that interacted with the 3′UTR of the *FMR1* gene did not change its expression in assayed cell lines, supporting the possibility that this miRNA is not involved in *FMR1* gene regulation in these cell lines, although this observation does not exclude the involvement of this miRNA in cells in patients or during a specific time of life.

## 4 Materials and methods

### 4.1 Cell cultures

To study miRNA expression, immortalized B lymphocytes of patients with FRAXopathies were used. Every cell line was obtained from one patient's blood with a specific diagnosis and was immortalized with Epstein-Barr virus. Cells were cultivated in RPMI 1640 GlutaMAX medium (Gibco, USA) supplemented with 15% fetal bovine serum (Gibco) and antibiotics. To analyze the interaction of miRNAs with the *FMR1* 3′UTR, HEK293A cells were cultured in DMEM/F12 (Biolot, Russia) supplemented with 10% fetal bovine serum (Gibco) and antibiotics. The cell lines GM04025, GM06865, GM06891, GM06891E, GM06897, and GM06895 were obtained from the Coriell Cell Repository (Coriell Institute, USA) and were described in an earlier study [31]. The cell lines CPG7, CPG8, CPG10, CPG18, CPG166, and HEK293A were obtained from the Cell Repository of the State Research Center of Virology and Biotechnology “Vector” (Koltsovo, Russia) and were genotyped as previously described [32].

### 4.2 FMR1 mRNA expression analysis

RNA was isolated from cell cultures using TRIzol Reagent (Thermo Fisher Scientific, USA) followed by reverse transcription using an M-MuLV-RH Reverse Transcription kit (Biolabmix, Russia) with random hexamer primers. Real-time PCR was performed with HS-qPCR SYBR Blue (2 × ) (Biolabmix) on a CFX96 Touch™ Real-Time PCR Detection System (Bio-Rad, USA). The sequences of the primers used in the present study are provided in Table S2. *FMR1* mRNA expression in the assayed cell lines was normalized based on the expression of the *FMR1* and GAPDH genes in the GM06895 cell line, as previously described, and all fold changes are presented as values relative to GM06895 [33,34]. The statistical significance of differences was calculated by a two-sample t-test as previously described [35]. Each sample consisted of three biological replicates assayed in a single experiment. Differences were significant if P < 0.05, where P is type I error.

### 4.3 FMRP protein level analysis

Each sample of cells was suspended in 100 μl of lysis buffer (50 mM Tris-HCl (pH 8), 150 mM NaCl, 1% NP-40, and 0.1% SDS) supplemented with Pierce™ Protease Inhibitor Mini Tablets (Thermo Fisher Scientific, USA). The resulting homogenates were centrifuged to remove insoluble precipitates, and the protein concentrations of the samples were determined using a Pierce™ BCA Protein Assay kit (Thermo Fisher Scientific, USA) according to the manufacturer's instructions. All samples were diluted with water to the same protein concentration. Protein extracts were collected and stored at −80 °C, and each sample was separated on a 10% gel by SDS-PAGE before being transferred to a PVDF transfer membrane (Thermo Fisher Scientific). Membranes were stained with Ponceau S to verify loading and transfer efficiency. FMRP and GAPDH were detected with anti-FMRP (ab130165, Abcam, USA) and anti-GAPDH (ab9485, Abcam) primary antibodies, respectively, and were detected using goat anti-mouse IgG Fc (A16084, Life Technologies, USA) and goat anti-rabbit HRP (AP187P, Sigma-Aldrich, USA) secondary antibodies, respectively. The maximum brightness of the FMRP protein signal in each cell line was determined using Image Studio Lite Ver 5.2 (LI-COR Biosciences, USA). To verify the significance of differences between groups, a two-sample t-test for independent samples was used. The differences between the experiments were considered significant when P < 0.05.

### 4.4 miRNA expression analysis

To evaluate the expression of the assayed miRNAs, primers were designed for both reverse transcription (RT) and real-time PCR (Table S1) using the stem-loop PCR method [36]. The same reverse primer Uni was used for all miRNAs since it is complementary to the primer sequence used for RT.

Total RNA was isolated from cell cultures using TRIzol reagent (Thermo Fisher Scientific) followed by reverse transcription with an M-MuLV-RH Reverse Transcription kit (Biolabmix). The reaction mixture contained 1 × RT-mix, reverse transcriptase (100 units), total RNA sample (500 ng) and a primer complementary to a specific miRNA (200 nM). The reaction was carried out in a C1000 touch thermal cycler (Bio-Rad) using the following protocol: 16 °C for 20 min, 42 °C for 40 min, and 70 °C for 10 min.

To analyze miRNA expression, 2 × BioMaster HS-qPCR reagent (Biolabmix) was used. In addition to the standard components, the reaction mixture contained 3 μl of the reverse transcription product, 0.2 μM labeled probe designed for each miRNA, 1.5 μM forward primer and 0.7 μM reverse primer. Each reaction was carried out in three biological and three technical repetitions. qPCR was performed in a CFX96 Touch™ Real-Time PCR Detection System (Bio-Rad) using the following protocol: 95 °C for 10 min, 95 °C for 15 s, and 60 °C for 1 min (steps 2–3, 39 cycles). The obtained data were analyzed using Bio-Rad CFX Manager 3.1 (Bio-Rad). The raw data were processed using the 2-ΔΔСt method according to the formula:

$$x=2^{-(\left(Ct1-Ct2\right)-\left(Ct3-Ct4\right))}$$

where Ct1 is the value of the threshold cycle of fluorescence of the studied miRNA in the studied cell line; Ct2 is the mean value of the threshold fluorescence cycle of two small nucleolar RNAs (SNORD48 and SNORD44) in the studied cell line; Ct3 is the value of the threshold fluorescence cycle of the studied miRNA in the cell line GM06895; and Ct4 is the mean value of the threshold cycle for SNORD48 and SNORD44 in the cell line GM06895. To verify the significance of differences between groups, a two-sample t-test for independent samples was used. The differences between the experiments were considered significant when P < 0.05.

### 4.5 Plasmid assembly for miRNA interaction analysis

The control plasmid (without an miRNA gene) was constructed from the following primary elements assembled in the indicated order: (i) a “CMV enhancer/promoter – rtTA-Advanced ORF – SV40 transcriptional terminator” cassette, (ii) a “CMV enhancer/promoter – FusionRed ORF – SV40 transcriptional terminator” cassette (on the complementary strand), (iii) a “hPGK promoter – eGFP ORF – 1790-bp *FMR1* 3’UTR” cassette, (iv) a tetracycline-inducible promoter – puromycin resistance ORF – SV40 transcriptional terminator, (v) a ColE1 origin of replication and (vi) an ampicillin resistance gene (on the complementary strand). This plasmid was used as a control for reporter protein fluorescence levels without miRNA influence. The experimental plasmids harbored the studied miRNA genes, which were cloned at the Bpu14I restriction site (TT^CGAA) located immediately after the puromycin resistance ORF in the control plasmid. Sequences of miRNA genes were amplified from GM06895 cell line DNA with Q5 polymerase (NEB, USA) and specific primers harboring restriction enzyme recognition sites (Table S3). All plasmid constructs were verified by Sanger sequencing. The complete nucleotide sequences of the plasmid without miRNA genes were deposited in GenBank (MT921016).

The plasmids were propagated in NEB Stable competent *E. coli* cells (NEB) under standard conditions. For transfection of HEK293A cells, plasmids were isolated and purified using a Plasmid Midiprep 2.0 kit (Evrogen, Russia).

### 4.6 Analysis of reporter protein fluorescence

HEK293A cells were transfected with the reporter plasmids using Lipofectamine 3000 reagent (Thermo Fisher Scientific) upon reaching 70% confluence of the monolayer in a 24-well plate according to the manufacturer's recommendations. The following day, doxycycline was added at a concentration of 1 μg/ml, and the cells were incubated for 48 h. This concentration and incubation time are optimal for miRNA synthesis induction [37,38]. Subsequently, the transfected cells were harvested from the plate using TrypLE Express Enzyme (Thermo Fisher Scientific), and fluorescence was measured in a dark, round-bottomed 96-well plate using an EnVision 2105 Multimode Plate Reader (PerkinElmer, USA). Background noise was determined by measuring the fluorescence signal in cells without a plasmid. For each construct carrying a specific miRNA, fluorescence was analyzed in three biological replicates. The change in eGFP fluorescence is presented as a dimensionless value (K) calculated according to the following formula: $K=\frac{\text{FRdox}}{\text{FR}}$, where FR_dox_ is the ratio of the eGFP to FusionRed fluorescence level 48 h after the addition of doxycycline and FR is the same ratio the day after transfection before the addition of doxycycline. The fluorescence analysis results for each plasmid with a specific miRNA are presented as the fold change relative to the fluorescence values in the control plasmid, calculated as follows: $X=\frac{\text{Kn}}{\text{Kcont}}$, where Kn is the value for a plasmid containing a specific miRNA.

## Declaration of competing interestCOI

The authors declare no conflict of interest.

## Funding

This work was supported by the 10.13039/501100006769Russian Science Foundation grant 16-14-10288 for plasmid construction and by the 10.13039/501100006769Russian Science Foundation grant 18-15-00099 for the rest of the study.

## CRediT authorship contribution statement

**Alexander A. Dolskiy:** Conceptualization, Formal analysis, Methodology, Writing - original draft, All authors have read and agreed to the published version of the manuscript. **Irina V. Grishchenko:** Formal analysis, All authors have read and agreed to the published version of the manuscript, All authors have read and agreed to the published version of the manuscript, Writing - original draft. **Natalya A. Lemskaya:** Resources, All authors have read and agreed to the published version of the manuscript. **Alexey V. Pindyurin:** Resources, All authors have read and agreed to the published version of the manuscript, Writing - original draft, All authors have read and agreed to the published version of the manuscript. **Lidiya V. Boldyreva:** Resources, All authors have read and agreed to the published version of the manuscript, Writing - original draft, All authors have read and agreed to the published version of the manuscript. **Vladimir O. Pustylnyak:** Resources, All authors have read and agreed to the published version of the manuscript, Writing - original draft, All authors have read and agreed to the published version of the manuscript. **Dmitry V. Yudkin:** Conceptualization, Writing - original draft, Project administration, All authors have read and agreed to the published version of the manuscript.

## Appendix A Supplementary data

Multimedia component 1 Multimedia component
1

Multimedia component 2 Multimedia component
2

Multimedia component 3 Multimedia component
3
