# Supplementary material for: miRNA expression and interaction with the 3′UTR of FMR1 in FRAXopathy pathogenesis
Source: Noncoding RNA Res. 2020 Dec 3;6(1):1–7. doi: 10.1016/j.ncrna.2020.11.006 (PMC7781359; doi:10.1016/j.ncrna.2020.11.006)
Supplement: Multimedia component 1 [file mmc1.docx]

**Supplementary table 1**. Primers for miRNA expression analysis

| miRNA | NCBI | miRBase | mirdb Target Score to 3'-UTR of *FMR1* mRNA | mature miRNA sequence | RT primer 5'->3' | Forward primer 5'->3' | Probe 5'->3' |
| --- | --- | --- | --- | --- | --- | --- | --- |
| hsa-miR-182-5p | NR_029614.1 | MIMAT0000259 | 87 | UUUGGCAAUGGUAGAACUCACACU | GTCGTATCCAGTGCAGGGTCCGAGGTATTCGCACTGGATACGACagtgtg | CAGCGGTTTGGCAATGGTAGA | (FAM)-CTGGATACGACAGTGTGAGTTC-(RTQ-1) |
| hsa-miR-23a-3p | NR_029495.1 | MIMAT0000078 | 85 | AUCACAUUGCCAGGGAUUUCC | GTCGTATCCAGTGCAGGGTCCGAGGTATTCGCACTGGATACGACggaaat | TCCGGATCACATTGCCAGGG | (FAM)-ACTGGATACGACGGAAATCCC-(RTQ-1) |
| hsa-miR-25-3p | NR_029498.1 | MIMAT0000081 | 94 | CAUUGCACUUGUCUCGGUCUGA | GTCGTATCCAGTGCAGGGTCCGAGGTATTCGCACTGGATACGACtcagac | ATGCGGCATTGCACTTGTCTC | (FAM)-ACTGGATACGACTCAGACCGA-(RTQ-1) |
| hsa-miR-148a-3p | NR_029597.1 | MIMAT0000243 | 96 | UCAGUGCACUACAGAACUUUGU | GTCGTATCCAGTGCAGGGTCCGAGGTATTCGCACTGGATACGACacaaag | TTGCGGTCAGTGCACTACAGA | (FAM)-CACTGGATACGACACAAAGTTCT-(RTQ-1) |
| hsa-miR-410-3p | NR_030156.1 | MIMAT0002171 | 87 | AAUAUAACACAGAUGGCCUGU | GTCGTATCCAGTGCAGGGTCCGAGGTATTCGCACTGGATACGACacaggc | CGGCGGAATATAACACAGATGG | (FAM)-ACTGGATACGACACAGGCCAT-(RTQ-1) |
| hsa-miR-139-5p | NR_029603.1 | MIMAT0000250 | 98 | UCUACAGUGCACGUGUCUCCAGU | GTCGTATCCAGTGCAGGGTCCGAGGTATTCGCACTGGATACGACactgga | GCGGTCTACAGTGCACGTG | (FAM)-ACTGGATACGACACTGGAGAC-(RTQ-1) |
| hsa-miR-221-3p | NR_029635.1 | MIMAT0000278 | 93 | AGCUACAUUGUCUGCUGGGUUUC | GTCGTATCCAGTGCAGGGTCCGAGGTATTCGCACTGGATACGACgaaacc | CGCGGAGCTACATTGTCTGC | (FAM)-ACTGGATACGACGAAACCCAG-(RTQ-1) |
| hsa-miR-302a-3p | NR_029835.1 | MIMAT0000684 | 79 | UAAGUGCUUCCAUGUUUUGGUGA | GTCGTATCCAGTGCAGGGTCCGAGGTATTCGCACTGGATACGACtcacca | TGGCGGTAAGTGCTTCCATGT | (FAM)-ACTGGATACGACTCACCAAAACA-(RTQ-1) |
| SNORD48 (U48) | NR_002745.1 |  |  | AGTGATGATGACCCCAGGTAACTCTGAGTGTGTCGCTGATGCCATCACCGCAGCGCTCTGACC | GTCGTATCCAGTGCAGGGTCCGAGGTATTCGCACTGGATACGACggtcag | GCGGAGTGATGATGACCCC | (FAM)-ACTGGATACGACGGTCAGAG-(RTQ-1) |
| SNORD44 (U44) | NR_002750.2 |  |  | CCTGGATGATGATAAGCAAATGCTGACTGAACATGAAGGTCTTAATTAGCTCTAACTGACT | GTCGTATCCAGTGCAGGGTCCGAGGTATTCGCACTGGATACGACAgtcagt | TCGGCCTGGATGATGATAAGC | (FAM)-ACTGGATACGACAGTCAGTTAGA-(RTQ-1) |
|  | | | | | | | |
| Universal pimer for miR expression analysis (Uni) | TCGTATCCAGTGCAGGGTCC |  |  |  |  |  |  |
