# Supplementary material for: miRNA expression and interaction with the 3′UTR of FMR1 in FRAXopathy pathogenesis
Source: Noncoding RNA Res. 2020 Dec 3;6(1):1–7. doi: 10.1016/j.ncrna.2020.11.006 (PMC7781359; doi:10.1016/j.ncrna.2020.11.006)
Supplement: Multimedia component 2 [file mmc2.docx]

**Supplementary table 1.** Primers for FMR1 mRNA expression analysis

| Primer | Sequence 5' -> 3' | Reference |
| --- | --- | --- |
| FMR1-F | GCTAAAGTGAGGATGATAAAG | NM_001185075.1 |
| FMR1-R | ATCCTTATGTGCCGCCTCTTTGG |  |
| EIF4A2-F | AGGGTCAAGTCGTGTTCTGAT | NM_001967.4 |
| EIF4A2-R | CCACACCTTTCCTCCCAAATC |  |
| GAPDH-F | GAGTCAACGGATTTGGTCGT | NM_001289745.3 |
| GAPDH-R | GACAAGCTTCCCGTTCTCAG |  |
