# Supplementary material for: miRNA expression and interaction with the 3′UTR of FMR1 in FRAXopathy pathogenesis
Source: Noncoding RNA Res. 2020 Dec 3;6(1):1–7. doi: 10.1016/j.ncrna.2020.11.006 (PMC7781359; doi:10.1016/j.ncrna.2020.11.006)
Supplement: Multimedia component 3 [file mmc3.docx]

**Supplementary table 3.** Primers for genetic construction

| Primer | Sequence 5' -> 3' | Reference |
| --- | --- | --- |
| pre-miR-182-gene-F | TAAGCATTCGAAATGGGCGCCTCTGTCCTG | NC_000007.14 |
| pre-miR-182-gene-R | TAAGCATTCGAAACCCAGCAGTCCGGGACC |  |
| pre-miR-23a-gene-F | TAAGCATTCGAAACCTCCTTTGCTCTCTCTCTC | NW_021160022.1 |
| pre-miR-23a-gene-R | TAAGCATTCGAAGGCTTCGGGGCCTCTCTG |  |
| pre-miR-25-gene-F | TAAGCATTCGAAGCTCCCCAAGCTCCATCTG | NC_000007.14 |
| pre-miR-25-gene-R | TAAGCATTCGAAGACACCCTTGTTCTGGCTTTA |  |
| pre-miR-410-gene-F | TAAGCATTCGAACTCACCTTTGATGTCCCATCC | NC_000014.9 |
| pre-miR-410-gene-R | TAAGCATTCGAATCAGGGGAAAGGATGACAAATG |  |
| pre-miR-139-gene-F | TAAGCATTCGAACGGAGCTGGTTGTGGGCG | NC_000011.10 |
| pre-miR-139-gene-R | TAAGCATTCGAACTCCTCCCTCTTTCTCTTCCT |  |
| pre-miR-221-gene-F | TAAGCATTCGAACTGTTGGTTTTCTTTTCCTTGTGG | NC_000023.11 |
| pre-miR-221-gene-R | TAAGCATTCGAACCTTTCTCTGCACTCTATTCAATG |  |
| pre-miR-302a-gene-F | TAAGCATTCGAACAAGCCAGCACACCTTTTGTTAC | NC_000004.12 |
| pre-miR-302a-gene-R | TAAGCATTCGAAGCTTAAATATATGAGCTGCGGTC |  |
| 3’-UTR- FMR1-F | ACTGCATAATTCTGAAGT | NC_000023.11 |
| 3’-UTR- FMR1-R | GCAGAGGAAGATCAAAC |  |
